# Initial Psychometric Testing and Validation of the Italian Version of the Canine Brief Pain Inventory in Dogs With Pain Related to Osteoarthritis

**DOI:** 10.3389/fvets.2021.736458

**Published:** 2021-09-17

**Authors:** Giorgia della Rocca, Alessandra Di Salvo, Cristina Medori, Maria Federica della Valle, Dottie Cimino Brown

**Affiliations:** ^1^Department of Veterinary Medicine, Research Center on Animal Pain (CeRiDA), University of Perugia, Perugia, Italy; ^2^Science Information and Documentation Center (CeDIS), Innovet Italia Srl, Saccolongo, Italy; ^3^Elanco Animal Health, Greenfield, IN, United States

**Keywords:** canine brief pain inventory, chronic pain, dog, osteoarthritis, Italian validation

## Abstract

The Canine Brief Pain Inventory (CBPI) is an owner-administered questionnaire, originally developed and validated in English, used to assess canine chronic pain in terms of severity and interference with daily life activities. The aim of the present study was to perform a preliminary validation of an Italian version of the CBPI. Translation was performed and the resulting questionnaire was administered to 45 native Italian speaking owners of dogs suffering from chronic pain due to radiographically confirmed osteoarthritis. Psychometric properties of the Italian CBPI including construct validity, convergent validity and reliability were evaluated. Construct validity was assessed by factor analysis and confirmed a two-factor model (i.e., pain severity and interference factors). The respective scores, that is, the pain severity score (PSS) and pain interference score (PIS), exhibited a substantial negative correlation with overall quality of life score. Pain severity and interference items showed a mean inter-item correlation of 0.90 and 0.80, respectively. For each question, communality ranged from 0.84 to 0.97, highlighting strong internal consistency and suggesting that PSS and PIS can be calculated by averaging the items contained within each factor. Cronbach's α was 0.97 and 0.96 for PSS and PIS, respectively. The present findings confirmed the main psychometric properties of the Italian version of the CBPI, providing clinicians and researchers with a useful metrology instrument to evaluate the severity of chronic pain and its interference with daily life activities in dogs with osteoarthritis owned by Italian speaking people. Further properties of the questionnaire need to be evaluated in future research and larger studies are warranted.

## Introduction

Pain is an unpleasant sensory and emotional experience accompanying a large variety of medical and surgical conditions, both in humans and animals.

One of the major challenges for veterinary practitioners is to recognize and assess pain, identify the pathogenesis and arrange a management plan as appropriate. Noteworthy, pets cannot verbalize their pain which can thus be evaluated only indirectly, either through (i) physical examination, with special reference to cardiovascular, respiratory and neurologic signs, in addition to musculoskeletal changes, (ii) administration of validated questionnaires to owners to quantify pain related behaviors or (iii) *ex juvantibus* criteria (diagnosis based on the animal response to analgesics). Particularly if used together, these tools may help the veterinary practitioner recognize if the animal is in pain and identify the most appropriate therapeutic approach (1).

Appropriately validated pain scales are extremely useful in the diagnostic *iter*, providing the observer (veterinary practitioner and/or animal owner) with reliable, ready-to-use tools.

In the last two decades, scoring systems to assess acute or chronic pain in dogs have been developed (2–5). Their validation was achieved through rigorous psychometric approaches, including item selection, questionnaire construction and testing for validity, reliability and responsiveness (6).

While some metrology instruments for pain assessment in animals have recently been validated in Italian (e.g., the short form of the Glasgow Composite Measure Pain Scale, the UNESP Botucatu Multi Composite Pain Scale and the UNESP Botucatu Unidimensional Composite Pain Scale) (1, 7, 8), no assessment tool for chronic pain in dogs is currently available in this language.

The Canine Brief Pain Inventory (CBPI) is a freely available, reliable tool to be administered to the owners of dogs with chronic pain associated with disorders such as osteoarthritis and bone cancer (3, 9–13). It is composed of 10 questions divided into two domains (i.e., pain severity and pain's interference with daily activities). In addition, there is a stand-alone final question to score the animal's quality of life (QoL), which does not contribute to the overall CBPI scoring. The Pain Severity Score (PSS) is obtained by averaging the results of the first four items (i.e., worst pain, least pain, average pain, current pain), while the results of further six items (i.e., interference with general activity, enjoyment of life, ability to rise, walk, run, climb) are averaged to give the Pain Interference Score (PIS).

As is the case with most pain scoring systems, the CBPI was originally developed in the English language, with Swedish and French versions being recently validated (14, 15). According to a recent survey by the British Council only about 12% of Italians have a good English language skills, which likely contributes to why pain-scoring systems are rarely used by Italian veterinarians for the assessment of pain (16). Accordingly, there is an important need to have a reliable translation of the CBPI to be administered to dog owners in Italy. Given their different origins, the English and Italian languages do not always have conceptually equivalent terms and specific translation challenges exist, especially in the attitude and behavior field. A rigorous and accurate process of translation and cultural adaptation of the questionnaire is thus required. Moreover, the psychometric features of the newly translated scale need to be evaluated, in order to ensure maintenance of meaning and content as well as structure and relevance of the scale (17–20).

The aim of the present study was to develop and perform initial psychometric testing of the Italian version of the CBPI, hypothesizing that it would have the same psychometric properties of the original English one. Construct validity via factor analysis and convergent validity between PSS/PIS and QoL were evaluated for validity assessment. Internal consistency using inter-item correlation matrices and Cronbach's α coefficient were evaluated to assess reliability.

## Materials and Methods

### Translation Procedure

One of the authors (GdR), fluent in the target language translated the English version of the CBPI into Italian. The translation was then reviewed for accuracy by three outside reviewers, all of whom are bilingual.

The Italian version of the CBPI is available as Supplementary Material.

### The Italian Canine Brief Pain Inventory

Like the original English CBPI, the Italian version consists of 4 questions addressing pain severity (worst pain, least pain, average pain, current pain) and 6 additional questions designed to assess pain's interference with the daily activities of the dog (general activity, enjoyment of life, ability to rise, walk, run and climb stairs). Owners answer each question on a 0–10 numerical rating scale (0 = no pain / no interference and 10 = extreme pain / complete interference). Pain Severity Score (PSS) and Pain Interference Score (PIS) are obtained by averaging the results obtained from each domain (severity and interference, respectively). Additionally, a last global QoL question is included in order to obtain the owner's overall impression of his/her pet's status. The latter item is scored on a 5-point categorical rating scale (poor, fair, good, very good, excellent) and does not contribute to the CBPI pain scores in the original questionnaire, nor in the Swedish, French or Italian translated versions.

### Patients and Study Design

The study included owners of dogs that had history, clinical signs and radiographic evidence consistent with painful appendicular osteoarthritis, presenting from January 2020 to January 2021. Sample size was chosen on the basis of the subject to item ratio between 4:1 and 5:1 (21).

Dogs with confounding comorbidities (i.e., pain arising from pathologies other than osteoarthritis) were not included in the patient recruitment. The owners were all native Italian speakers and provided informed consent to participate in the study. Each owner completed a single administration of the Italian CBPI questionnaire after having been instructed on its use. This study is part of research evaluating the efficacy and tolerability of a therapeutic protocol for osteoarthritis pain, which was approved by the Local Ethical Committee of the University Perugia, Italy (protocol n. 2018-13).

### Statistical Analysis

The construct validity of the Italian CBPI was assessed by factor analysis which identifies how many dimensions (*dimensionality*) are addressed in the tool, and establishes the relationship between the instrument items (questions) and each dimension (6, 22, 23). Loading values ≥ 0.32 are considered indicative of consistency between the question and the theoretical factor (24). The results of all items except the last one (the overall dog's QoL) were entered into an orthogonal, varimax-rotated factor analysis and the eigenvalue (variance of the factor) was calculated (21).

For the analysis of convergent validity (i.e., the extent of correlation between two measures of a construct that theoretically should be related) (25), Pearson correlation coefficient was used, with the correlation of PSS and PIS being evaluated with respect to QoL score. Week, moderate and strong correlation was considered with Rho <0.35, from 0.36 to 0.67, and from 0.68 to 1.0, respectively (26).

The reliability of the Italian CBPI was assessed through evaluating its internal consistency, which verifies the interrelations among the different items of the questionnaire (6). Inter-item correlation matrices were evaluated and Cronbach's coefficient α was calculated for PSS and PIS, with a cut-off value of at least 0.80 for each set of questions (PSS and PIS) to be considered a reliable scale (6). In addition, to determine the proportion of the variance for each question that could be explained by the PSS or PIS factor, communality was evaluated. Communality values ≤ 0.40 would indicate that questions are not related to each other, or additional factors need to be evaluated (21).

Tests for skewness and kurtosis were used to evaluate the distribution of continuous variables dog age, dog weight, PSS and PIS.

All analyses were performed using Stata 16 (Statacorp LP, College Station TX, USA).

## Results

### Patient Characteristics

Forty-five dogs with pain related to osteoarthritis participated in this study. Twenty-one were females (3 intact, 18 neutered) and 24 males (18 intact, 6 neutered). The mean age and mean weight (± standard deviation) of the dogs was 8.7 years (± 3.0 years) and 28.7 kg (± 11.3 kg), respectively. The dogs comprised 16 different breeds: 15 Labrador Retriever; 8 Mixed Breed; 3 each Border Collie, Cane Corso, Golden Retriever; 2 each Chow Chow, German Shepherd and 1 each Australian Shepherd, English Springer Spaniel, Maremma Sheepdog, Pekingese, Pitbull, Rottweiler, Scotch Collie, Staffordshire Bull Terrier, and Toy Poodle.

Dog's age and weight, as well as PSS and PIS resulted normally distributed on the basis of the applied tests (overall test statistic > 0.05).

### Questionnaire Results

Table 1 shows the results (reported as mean and standard deviation, minimum and maximum) obtained for each item of the questionnaire.

**Table 1:** Results for each item of the Italian Canine Brief Pain Inventory (expressed as mean, standard deviation, minimum and maximum) obtained from 45 owners of dogs with osteoarthritis.

**Item**	**Mean**	**Std. Dev**.	**Min**	**Max**
Worst pain	4.44	1.98	1	8
Least pain	2.69	1.95	1	7
Average pain	3.60	1.99	1	8
pain know	3.11	2.30	0	8
Activity	3.80	2.90	0	10
Enjoyment	3.02	2.68	0	10
Rising	4.02	2.44	0	9
Walking	3.67	2.61	0	9
Running	4.64	3.18	1	10
Climbing	4.51	2.83	1	10

### Construct Validity

A two-factor model was confirmed when construct validity was assessed by factor analysis. Indeed, two factors were identified with an eigenvalue > 1.0. Those two factors made up 88% of the variance. All other factors showed eigenvalues < 0.65.

Pain severity and interference scores resulted in factor loadings ranging from 0.76 to 0.80 and from 0.39 to 0.75, respectively (Table 2). Hence, factor loading of all questions exceeded the 0.32 loading value cut-off, confirming that each question is associated with the factor.

**Table 2:** Factor loadings and communality of each item of the Italian Canine Brief Pain Inventory. Cronbach's α coefficient and average inter-item correlation of the severity of pain domain and pain interference with function domain, as well as the total instrument.

**Factor and items**	**Factor loadings**	**Communality**	**Cronbach's**	**Average inter-item correlation**
**Severity of pain**			**0.97**	**0.90**
Item 1: Pain at its worst	0.79	0.83	0.98	0.94
Item 2: Pain at its least	0.78	0.96	0.88	0.88
Item 3: Pain at its average	0.80	0.97	0.87	0.87
Item 4: Pain right now	0.76	0.91	0.90	0.90
**Pain interference**			**0.96**	**0.80**
Item 5: General activity	0.44	0.88	0.95	0.79
Item 6: Enjoyment of life	0.39	0.85	0.96	0.81
Item 7: Ability to rise to standing	0.48	0.84	0.95	0.81
Item 8: Ability to walk	0.49	0.91	0.95	0.78
Item 9: Ability to run	0.72	0.88	0.95	0.79
Item 10: Ability to climb stairs	0.75	0.84	0.95	0.81
**Total instrument**			**0.98**	**0.81**

*Cronbach's α coefficient and average inter-item correlation of the severity of pain domain and pain interference with function domain, as well as the total instrument*.

### Convergent Validity

**Figure 1:**
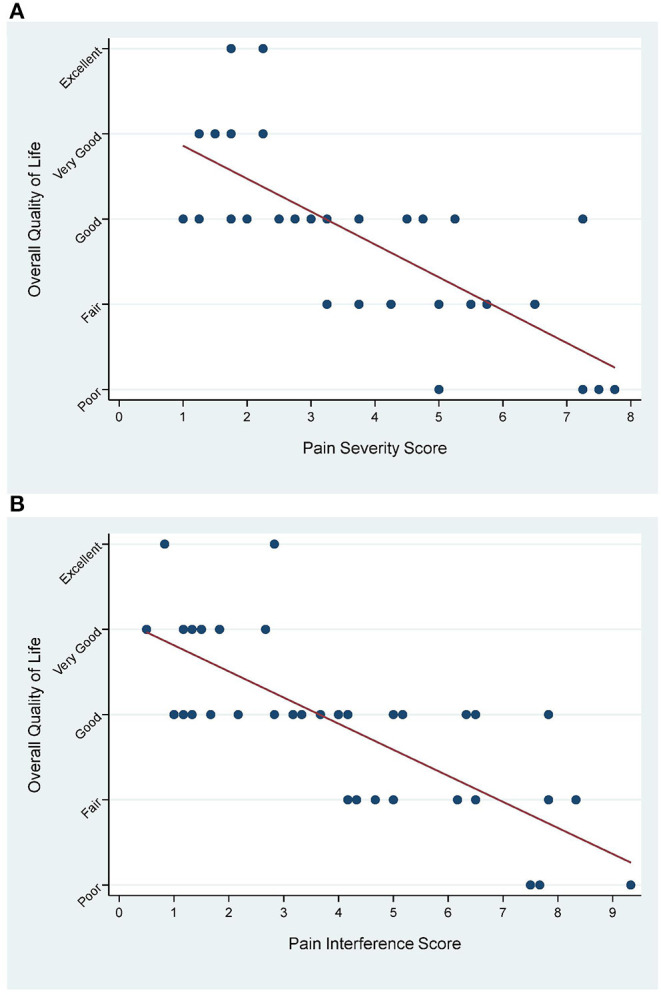
Pearson Correlation between PSS (**A**; *r* = −077) and PIS (**B**; *r* = −078) with the overall QoL question (*p* < 0.001 for both).

There was a strong negative correlation between the PSS (Figure 1A; *r* = −077) and PIS (Figure 1B; *r* = −078) with the overall QoL, confirming that increasing pain severity and pain interference with daily life activities was associated with decreased QoL. Results were statistically significant for both factors (*p* < 0.001).

### Reliability

There were no negative inter-item correlations. The mean inter-item correlation for PSS and PIS were 0.90 and 0.80, respectively (Table 2).

For all questions, communality ranged from 0.84 to 0.97, being well-above the 0.40 cut-off value (Table 2), thus highlighting strong internal consistency (i.e., PSS and PIS are calculated by averaging the items contained within each factor, as they measure the same concept). The Cronbach's α coefficient calculated for PSS and PIS was 0.97 and 0.96, respectively (Table 2), well above the 0.80 cut-off value, thus confirming the reliability of the translated CBPI.

## Discussion

Worldwide, veterinary practitioners are increasingly expected to play a proactive role in diagnosing and managing animal's pain. The development of metrology instruments for pain assessment supports this movement. Importantly, the availability of reliable questionnaires to measure chronic pain in different mother-tongue countries offers veterinary practitioners, dog owners, as well as researchers key instruments to assess and appropriately manage chronic pain. Moreover, it enables comparison of data collected across local as well as international arenas. Indeed, well-validated questionnaires need to be translated and validated in a variety of languages in order to allow their use in daily veterinary practice and/or clinical trials.

A reliable Italian version of the well-known CBPI gives Italian speaking owners, veterinary practitioners, as well as researchers a validated tool to quantify the severity of pain and its impact on daily living activities in dogs with chronic pain, such as those affected with OA.

In the present study, the initial validation of the Italian CBPI consisted in the application of psychometric tests aimed to confirm the two-factor model, convergent validity, and the internal consistency originally demonstrated for the English version.

With regard to construct validity, the factor analysis confirmed a two-factor structure of the Italian CBPI (i.e., pain severity factor and pain interference factor), consistent with what was previously found for the English as well as the French version of the questionnaire (10, 15). Notably, the factor loading for every item of the Italian CBPI resulted higher than the established cut-off value (0.32), while the factor loading for one of the questions in the pain interference domain of the French version (i.e., enjoyment of life) fell below (15). It follows that the Italian CBPI has the same number of factors and the same questionnaire structure as the original English version. This cannot be assumed and indeed did not hold true for the Swedish translation, in which three items—all intended to score pain interference with daily activities (i.e., general activity, enjoyment of life and ability to rise)—loaded equally on the two extracted factors, indicating they might assess pain interference or pain severity without distinction (14).

Consistent with the English and French versions (10, 15), the Italian CBPI showed a strong negative correlation between pain scores (i.e., PSS and PIS) and the overall QoL score, thus confirming that higher pain is related to poorer dog's quality of life (convergent validity).

With respect to reliability, the Italian CBPI did not show any difference from the previously validated versions, with no negative inter-item correlations being detected and high communality identified for all items.

In conclusion, the Italian translation of the CBPI is a psychometrically sound tool for use by Italian mother-tongue owners, veterinarians and researchers, given the strong validity and reliability results shown in the present study.

Nevertheless, one should not ignore that questionnaire validation is an ongoing process (27). In line with what has been done for the original English CBPI, further psychometric properties need to be evaluated in future studies (e.g., construct validity by hypothesis testing, criterion validity, responsiveness, reproducibility and stability), with multiple administrations of the questionnaire and larger sample sizes (3, 10, 12, 13). Finally, further studies are warranted to assess whether current results may also apply to dogs with chronic pain of different origins, such as bone cancer (11).

With all of this in mind, the present study provides preliminary validation of the Italian version of the CBPI through confirming the main psychometric properties of the pain scale (i.e., construct and convergent validity as well as reliability). Hopefully, this will foster a wider use of CBPI among Italian speaking veterinary practitioners and researchers, in order to measure and monitor chronic pain in osteoarthritis dogs.

## Data Availability Statement

The raw data supporting the conclusions of this article will be made available by the authors, without undue reservation.

## Ethics Statement

The animal study was reviewed and approved by Local Ethical Committee of the University Perugia, Italy. Written informed consent was obtained from the owners for the participation of their animals in this study.

## Author Contributions

GdR: study design, data collection, and preparation of the manuscript. ADS: critical revision of the manuscript. CM: data collection. MFdV: study design and preparation of the manuscript. DCB: statistical analysis, data interpretation, and preparation of the manuscript. All authors contributed to the article and approved the submitted version.

## Conflict of Interest

MFdV is cofounder and consultant for Innovet Italia Srl; CM is employed by Innovet Italia srl; DCB is employed by Elanco Animal Health, Greenfield IN, USA. The remaining authors declare that the research was conducted in the absence of any commercial or financial relationships that could be construed as a potential conflict of interest.

## Publisher's Note

All claims expressed in this article are solely those of the authors and do not necessarily represent those of their affiliated organizations, or those of the publisher, the editors and the reviewers. Any product that may be evaluated in this article, or claim that may be made by its manufacturer, is not guaranteed or endorsed by the publisher.
