# Supplementary material for: Initial Psychometric Testing and Validation of the Italian Version of the Canine Brief Pain Inventory in Dogs With Pain Related to Osteoarthritis
Source: Front Vet Sci. 2021 Sep 17;8:736458. doi: 10.3389/fvets.2021.736458 (PMC8484962; doi:10.3389/fvets.2021.736458)
Supplement: Supplementary file 1 [file Data_Sheet_1.PDF]

Data __/__/____ Paziente _______________

QUESTIONARIO PER LA VALUTAZIONE DEL DOLORE PERSISTENTE NEL CANE - CANINE BRIEF PAIN INVENTORY (CBPI)

Descrizione del Dolore:

Valuta il dolore del tuo cane.

- Seleziona la casella vicino al numero che meglio descrive il livello del peggior dolore provato negli ultimi 7 giorni.

☐0 ☐1 ☐2 ☐3 ☐4 ☐5 ☐6 ☐7 ☐8 ☐9 ☐10

Nessun Dolore … **Dolore Estremo**

- Seleziona la casella vicino al numero che meglio descrive il livello del dolore più lieve provato negli ultimi 7 giorni.

☐0 ☐1 ☐2 ☐3 ☐4 ☐5 ☐6 ☐7 ☐8 ☐9 ☐10

Nessun Dolore … **Dolore Estremo**

- Seleziona la casella vicino al numero che meglio descrive il livello del dolore di intensità media provato negli ultimi 7 giorni.

☐0 ☐1 ☐2 ☐3 ☐4 ☐5 ☐6 ☐7 ☐8 ☐9 ☐10

Nessun Dolore … **Dolore Estremo**

- Seleziona la casella vicino al numero che meglio descrive il livello del dolore provato in questo momento.

☐0 ☐1 ☐2 ☐3 ☐4 ☐5 ☐6 ☐7 ☐8 ☐9 ☐10

Nessun Dolore … **Dolore Estremo**

Descrizione della Funzionalità:

Seleziona la casella vicino al numero che descrive quanto il dolore ha interferito negli ultimi 7 giorni con:

- Attività Generale.

☐0 ☐1 ☐2 ☐3 ☐4 ☐5 ☐6 ☐7 ☐8 ☐9 ☐10

Non Interferisce … **Interferisce Completamente**

- Gioia di vivere.

☐0 ☐1 ☐2 ☐3 ☐4 ☐5 ☐6 ☐7 ☐8 ☐9 ☐10

Non Interferisce … **Interferisce Completamente**

- Capacità di alzarsi da steso.

☐0 ☐1 ☐2 ☐3 ☐4 ☐5 ☐6 ☐7 ☐8 ☐9 ☐10

Non Interferisce … **Interferisce Completamente**

- Capacità di camminare.

☐0 ☐1 ☐2 ☐3 ☐4 ☐5 ☐6 ☐7 ☐8 ☐9 ☐10

Non Interferisce … **Interferisce Completamente**

- Capacità di correre.

☐0 ☐1 ☐2 ☐3 ☐4 ☐5 ☐6 ☐7 ☐8 ☐9 ☐10

Non Interferisce … **Interferisce Completamente**

- Capacità di salire le scale o saltare muretti.

☐0 ☐1 ☐2 ☐3 ☐4 ☐5 ☐6 ☐7 ☐8 ☐9 ☐10

Non Interferisce … **Interferisce Completamente**

Impressione Complessiva:

- Seleziona la casella vicino alla risposta che meglio descrive la qualità di vita del tuo cane nel complesso negli ultimi 7 giorni.

☐ **Scarsa** ☐ **Normale** ☐ **Buona** ☐ **Molto Buona** ☐ **Eccellente**
